# A Systematic Review of the Effectiveness of Dry Needling in Subacromial Syndrome

**DOI:** 10.3390/biology11020243

**Published:** 2022-02-04

**Authors:** María Blanco-Díaz, Rubén Ruiz-Redondo, Isabel Escobio-Prieto, Marta De la Fuente-Costa, Manuel Albornoz-Cabello, José Casaña

**Affiliations:** 1 Faculty of Medicine and Health Sciences, University of Oviedo, 33006 Oviedo, Asturias, Spain; blancomaria@uniovi.es (M.B.-D.); rubenerre4@gmail.com (R.R.-R.); marta@delafuentecosta.es (M.D.l.F.-C.); 2 Department of Physiotherapy, Faculty of Nursing, Physiotherapy and Podiatry, University of Sevilla, 41009 Sevilla, Spain; malbornoz@us.es; 3 Exercise Intervention for Health Research Group (EXINH-RG), Department of Physiotherapy, University of Valencia, 46010 Valencia, Spain; jose.casana@uv.es

**Keywords:** dry needling, subacromial syndrome, systematic review, metanalysis

## Abstract

**Simple Summary:**

Dry needling, used by physical therapists, is a treatment modality used for the management of musculoskeletal pain. It is a technique in which a fine needle is used to penetrate the skin, subcutaneous tissues and muscles, with the aim of mechanically disrupting the inner tissues. This technique is called dry needling as the procedure does not involve the injection of any substance. Subacromial syndrome is defined as any kind of non-traumatic, usually unilateral, shoulder problem that causes pain around the acromion, that usually gets worse during or after lifting the arm. It should not be treated with surgical methods as the first option, but with different physiotherapy techniques. In this review, an overview of the effects of dry needling combined with conventional physiotherapy in patients with subacromial syndrome is presented. One of the key focal points is that dry needling combined with physiotherapy is effective and safe in reducing the pain and disability caused by this pathology.

**Abstract:**

Our aim was to evaluate the effectiveness of dry needling (DN) combined with conventional physiotherapy in the recovery of patients with subacromial syndrome (SAS). A search was made of the main open access health science databases. The publication date was not limited for systematic reviews but was for randomized clinical trials (RCTs), which were limited to the last five years (from 2016) in English or in Spanish. Ninety-four studies were selected. In order to assess the quality of the studies, the JADAD scale or Oxford quality scoring system was used. A total of 402 patients were analyzed in all the studies in which the application of conventional physiotherapy was compared to the DN, either in a combination or in isolation. Improvements were obtained in pain intensity (Visual Analogic Scale—VAS), Range of Movement (ROM), Pressure Pain Threshold (PPT), functionality with Disabilities of the Arm, Shoulder and Hand (DASH) and the Shoulder Pain and Disability Index (SPADI), and in the cost–benefit ratio. DN is effective and safe in reducing the pain and disability produced by SAS, with the best combination of treatment turning out to be conventional physiotherapy together with DN, obtaining more stable and longer-lasting benefits than merely applying the techniques in isolation.

## 1. Introduction

Shoulder pain (SP) is the most common of the pathological conditions included in rotator cuff (RC) disease [1], with a prevalence in Spain of between 46–467 cases/100,000 inhabitants [2]. In 1972, Neer described the concept of Subacromial Pain Syndrome (SAPS) as “Pain in the anteromedial portion of the shoulder, secondary to impingement of the acromion on the rotator cuff and humeral head” [3]. Other more current authors such as Diercks et al. have defined this syndrome as “Shoulder problems causing pain, localized around the acromion, which often worsens while or after elevating the arm”. Under this name, the diagnosis of rotator cuff tendinopathy, subacromial bursitis, partial rotator cuff tear, biceps tendinopathy, and calcific tendinitis are classified as SAPS [4]. The most common presentation of SAPS is frozen shoulder (adhesive capsulitis), rotator cuff tendinopathy (RCT), and myofascial trigger points (MTP). The complex pathomechanical presentation and a lack of sensitivity and specificity in specific tests pose a serious challenge to making a correct diagnosis [5].

The primary presentation of shoulder impingement occurs when the RC tendons, long head of the biceps, glenohumeral capsule, and/or subacromial bursa get trapped between the humeral head and the acromion. In turn, secondary shoulder impingement is defined as a relative decrease in the subacromial space due to glenohumeral instability or abnormal function of the scapulothoracic articulation. This musculoskeletal condition presents in multiple forms, ranging from inflammation and degeneration of these structures to a complete tear of the RC tendons and a degenerative disease of the articulations of the shoulder girdle [1,2].

The characteristic clinical features of SAPS are nocturnal pain located in the anteromedial part of the shoulder, presenting as shoulder stiffness that may radiate to the lateral part of the arm and elbow and increase when raising the shoulder above 60° [3], and a limited range of motion that restricts daily living activities [6]; subacromial crepitation and weakness [4] are also symptoms frequently associated with this pathology.

Conservative treatment is the main option for patients with SAPS; however, the most appropriate strategy, the ‘Gold standard’, remains an unknown quantity. In fact, different interventions including injections, medication, exercise, electrotherapy, or even cognitive therapy are recommended, with different levels of evidence as referred to in clinical guidelines [5]. Treatment usually begins with conservative therapies (physical therapy, anti-inflammatory drugs and corticosteroid injections). However, a multimodal treatment plan including techniques such as stretching, manual therapy, mobilization techniques, applying cold packs, home exercise, ischemic compression of MTP in the shoulder muscles, ergonomic recommendations and dry needling (DN; intramuscular stimulation, medical acupuncture), have shown benefits in current studies [6]. DN, which is a treatment modality that is minimally invasive, cheap, easy to learn with appropriate training and carries a low risk [7], with the most common adverse effects being bruising, bleeding, and pain during or after treatment [8]. Injections into myofascial trigger points have been proposed by Travell and Simons [9]. The wider use of dry needling started after Lewit’s publication [10], where it was emphasized that the needling effect is distinct from that of the injected substance [7].

Despite the high prevalence of this pathology—between 7–26% of the general population, and a lifetime probability of suffering from this condition of around 67%—there are no systematic reviews (SR) in the scientific literature that evaluate the treatment, pain management, and clinical effectiveness of DN in SAPS. Hence, through this study, randomized clinical trials (RCTs) were reviewed in the literature in order to determine the effectiveness of DN treatment combined with traditional physiotherapy in the treatment and pain management of SAPS [11].

## 2. Materials and Methods

### 2.1. Search Strategy

This SR followed the Preferred Reporting Items for Systematic Reviews and Meta-Analyses (PRISMA) guidelines [12]. The protocol was registered in the International Prospective Register of Systematic Reviews (PROSPERO/NHS)—number CRD42021271574 [12,13]. Systematic research using Medline (via PUBMED), SCOPUS, WEB OF SCIENCE (WOS), PEDRo, COCHRANE LIBRARY and TRIPDATABASE was performed to identify trials suitable for inclusion in this SR. Keywords for the literature search were selected, with the authors’ agreement, using the PICOS approach: (P—participants; I—interventions; C—comparison; O—outcomes; and S—study design [13]. The terms used as key words are listed in Appendix A.

### 2.2. Selection Criteria

Two researchers (M.B.-D., R.R.-R.) independently reviewed the articles found (title and abstract screening, and full text) and a third author (M.F.-C.) acted as a referee in uncertain cases. In order to formulate the objective and the question of the review, the PICOS strategy was used [13] in which P = adults with unilateral shoulder pain of non-traumatic origin, for a duration of at least three months, pain intensity of at least four points on the VAS scale and diagnosed with SAPS. Gender was irrelevant. There were no criteria for height or body mass index (BMI); I = DN combined with conventional physiotherapy; C = control group that received no intervention or received standard/usual care, sham or placebo intervention; O = variables related to clinical outcomes as well as health-related quality of life (Table 1); and S = randomized controlled clinical trials, systematic reviews, and meta-analyses. This strategy enabled the establishment of critical reasoning on issues [13], and the formulation of the following question: “What is the existing scientific evidence on the treatment of adults diagnosed with SAPS through procedures of DN treatment combined with traditional physiotherapy”.

### 2.3. Measures

The variables used to assess the inputs, results and effects were variables related to patient data, clinical outcomes and health-related quality of life (Table 1).

All of the studies evaluated pain or pain-related outcomes. These were measured using a visual analog scale (VAS) [14], which consists of a straight line, with the endpoints defining extreme limits such as ‘no pain at all’ and ‘pain as bad as it could be’. Another numerical rating scale used (NRS) asks patients to circle the number between 0 and 10, 0 and 20 or 0 and 100 that best matches the intensity of their pain. It therefore only allows a less subtle distinction of pain levels compared to the VAS, where there is a theoretically unlimited number of possible responses [15].

Measurement of health-related quality of life is essential in the assessment of pain management outcomes and was measured using the Societal costs and Health-Related Quality of Life (EuroQol-D5) [16] and Quality-Adjusted Life-Year (QALY) questionnaires. QALYs are measures of health outcomes used in economic evaluations to capture changes in both the quantity and quality of life due to health interventions [17].

Deep muscular tissue sensitivity was measured using a pressure algometer and the Pressure Pain Threshold (PPT). This test determines the amount of pressure over a given area in which a steadily increasing nonpainful pressure stimulus turns into a painful pressure sensation [18].

Shoulder assessment: the majority of the studies measured functional status using the Disabilities of the Arm, Shoulder and Hand (DASH) scale [19], Penn Shoulder Score (PSS) [20], Global Rating of Change functional outcome score [21] (GROC) and Shoulder Pain and Disability Index functional outcome measure (SPADI) [22].

Function: measured by Range of Motion (ROM) testing procedures, Patient Specific Functional Scale (PSFS) [23], scapular dyskinesia and infraspinatus muscle function.

### 2.4. Data Sources

A systematic search of the following databases was performed for articles published up to December 16, 2021. Two researchers independently (MBD, RRR) conducted an electronic literature search (up to date day) on Medline (via PubMed), SCOPUS, Web of Science, PEDro, Cochrane Library and Trip Database using the same methodology. Titles, abstracts, and full-text papers were screened and assessed to identify eligible articles, with IEP, MAC and JC acting as arbiters. Details of the study participants, type of interventions, outcomes, and other information were extracted using a standardized data extraction form that included: study design, eligibility and exclusion criteria, duration of follow-up, randomization, blinding, number and characteristics of patients, type of treatment. This bibliographic review was carried out in two phases: The first (I) consisted of an analysis of the SR in relation to the aim of the study, in order to analyze the motivation behind performing the review. In the second phase (II), a review of the most relevant RCTs was performed.

### 2.5. Exclusion Criteria

By way of exclusion criteria: all articles not published in English or Spanish; studies conducted in patients with associated underlying pathology; surgery; treatments where DN not carried out in combination with conventional physiotherapy; narrative or nonsystematic reviews; all documents not aligned with the research problem. The bibliographic research focused on all articles published from 2016 to 2021 for RCTs, with no end date for the SRs.

### 2.6. Data Extraction

**Figure 1:**
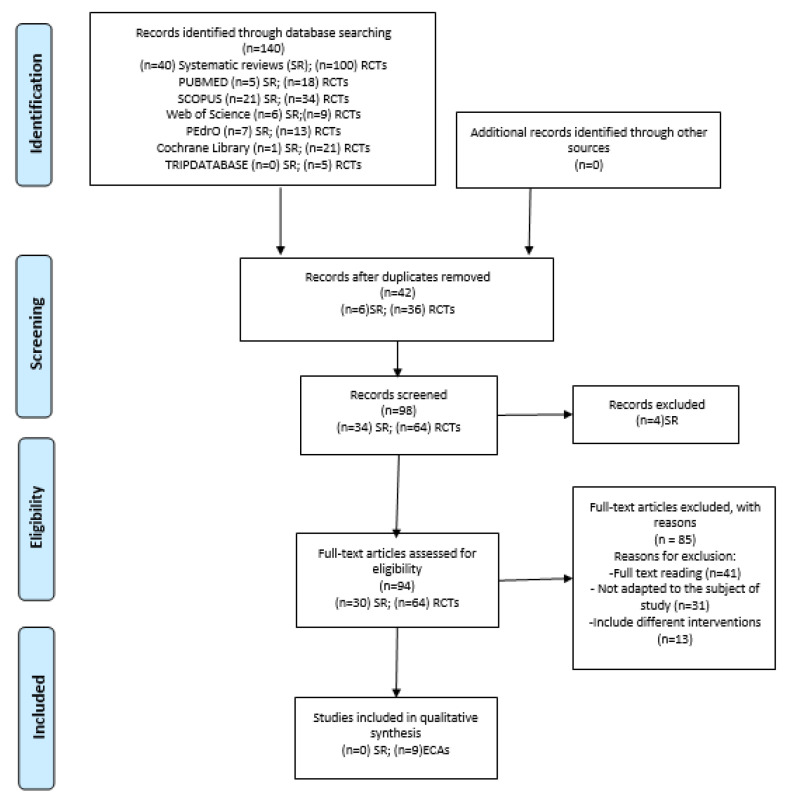
Flow chart of this systematic review.

After searching different keywords (Appendix A) in the aforementioned list of databases and sorting articles by title and summary, relevant articles were identified for complete reading, duplicate articles were eliminated, and the inclusion and exclusion criteria were applied to the sample of definitive data (Figure 1).

### 2.7. Methodological Quality Assessment

We evaluated the methodological quality and internal validity of the studies using the PEDro scale. The PEDro scale (0–10) is based on the Delphy list developed by Verhagen et al. [24]. Two independent evaluators (IEP and MAC) used the PEDro checklist to score each study. A study with a score of 4–5 was considered poor or acceptable, where a core below 4 was considered to indicate low methodological quality. Studies with a score below 6 were considered as having low or level 1 evidence, where a study with a score of 6–8 was considered good and a study with a score of 9–10 was considered excellent.

## 3. Results

### 3.1. Selection of Studies

The initial search in the databases gathered a total of 140 articles, 40 SRs, and 100 RCTs: 23 from PUBMED; 55 from SCOPUS; 15 from WOS; 20 from PEDro; 22 from the Cochrane Library and 5 from the Trip Database.

The initial screening phase produced 98 articles after removing duplicates (*n* = 42). Subsequently, all titles and abstracts were screened for eligibility in a standardized manner by two researchers (M.B.-D. and R.R.-R.). Any disagreement between the two reviewers was resolved by consensus and the arbitrator (M.F.-C.) was consulted to settle it. Finally, a total of four articles were removed.

The 94 remaining articles were screened for full textual review by two researchers; reasons for exclusion were registered. After full-text reading, 41 were excluded, because they were not suitable for the subject of the study (*n* = 31), or for including different treatment interventions (*n* = 13).

Figure 1 illustrates the different phases of the review, using an eligibility and data-synthesis PRISMA flow diagram.

### 3.2. Quality of Studies Included

Finally, nine RCTs [25,26,27,28,29,30,31,32,33] were included due to their meeting the inclusion criteria (Figure 1). Using the PEDro scale [14], three studies received a score of 10 and three scored 9; two studies received a score of 7 and one received a score of 6. These studies were considered “good”. Criteria and scoring are depicted in Table 2.

Due to the paucity of systematic reviews supporting the research question, a search for original studies (RCTs) was conducted.

In terms of the SR, as a result of the initial search, 40 reviews were detected. They were analyzed and accepted or discarded following the PRISMA criteria—resulting in 6 reviews being ruled out for seeming to be duplicates, 4 because their study was not identified as a “systematic review or metanalysis”, and 30 because they did not fit the study topic and did not answer the research question.

In order to assess the quality of the studies, the JADAD scale or Oxford quality scoring system [34] was used (Table 3), which scores five items in which randomization, blinding, and attrition rate are assessed. The scores range from zero (very poor) to five (rigorous). For this review, three points was established as the minimum requirement to be accepted and reviewed.

Two reviewers (I.E.-P, M.A.-C) independently performed the quality assessment, using PEDro and JADAD scales. An arbitrator (J.C.) was consulted to settle any disagreements.

### 3.3. Study Characteristics

Table 4 shows a summary of the characteristics of the nine RCTs selected for this SR.

In all the studies included, there was a total of 402 patients with SAPS, varying in range from 39 in the smallest sample size study [32] to 60 patients in the largest [35]. At the beginning of each study, the distribution between the groups selected was done in the fairest possible way, with all the groups having the same number of patients or with an intergroup variance of +1/−1.

Following the objective of this SR, it was mainly studies comparing conventional physiotherapy (manual therapy, therapeutic exercise, electrotherapy) with the addition of dry needling that were analyzed.

The general inclusion criteria for the studies analyzed that fulfilled the specificity criterion were unilateral shoulder pain of non-traumatic origin, shoulder pain of at least three months’ duration, intensity of pain of at least four points on the VAS scale and diagnosis of Subacromial Pain Syndrome (SAPS). One study assessed the effect of DN in patients whose main criterion was their status of being recently operated on for stabilization of the shoulder. Pregnant ladies, concomitant infections and cardiovascular pathology were excluded [32].

By way of exclusion criteria: the appearance of bilateral symptoms, history of prior shoulder fractures or luxation, diagnosis of cervical radiculopathy, interventions with corticosteroids, fibromyalgia, systemic illnesses such as multiple sclerosis, neck or shoulder surgery, any type of surgical intervention in the last year and a half and fear of needles.

As for the SAPS diagnosis, all the studies followed the clinical practice guidelines of the Dutch Orthopaedic Association [36], combining various orthopedic tests related to the RC in order to determine the pathology. Imani et al. [30] described being positive in both the Hawkins–Kennedy test and in the Neer sign and having a pattern of referred pain in the infraspinatus muscle (ISP) as inclusion criterion for patients, based on another of the analyzed studies [27,28,29]; physiotherapeutic diagnosis was the only diagnostic criterion that Kamali et al. followed [25] to diagnose SAPS.

With respect to demographic criteria, most of the studies chose a sample of patients between 18 and 65 years of age. Imani et al. limited age to a maximum of 55 years on the basis that from that age onwards, age-related alterations begin to appear (alterations in the posterior tilt angle and axillary swing in abduction of 90°). There were no criteria for gender, height, or even BMI [29].

Methodologically, six of the studies analyzed used an independent researcher, which endorses a correct data collection procedure [25,28,29,30,32,33], while in the other articles [27,31,36]—either due to the type of study or a lack of resources—the data were collected by the very researchers involved in the therapeutic intervention. Likewise, because of the characteristics of the intervention, as the patients were able to identify which treatment they were receiving, all the studies were described as ‘simple blind’.

In eight of the studies [26,27,28,29,30,31,32,33], one or several physiotherapy techniques were compared either in combination with or in isolation from DN, and one of the articles [25] compared DN techniques for their suitability in the treatment of patients—the intervention outline can be observed in Table 3. The results are quite revealing as, despite it not being an objective of this review, it is demonstrated that the application of physiotherapy in the treatment of SAPS is effective in practically 100% of the 402 patients analyzed, whether in combination with or in isolation from DN.

Thus, it remains to fulfill the objective of the study and find out whether clinical improvement is greater when applying DN or not. Arias-Buría et al. [28,29], who compared the application of therapeutic exercise (TE) with TE + DN, concluded that a combined intervention with DN and physiotherapy does result in significant differences between groups based on the DASH questionnaire (Disabilities of the Arm, Shoulder and Hand), declaring a statistically significant difference between the CG and EG (*p* < 0.001) in favor of the group that was given DN, with the following timing: in both the measurements at 1 week post-intervention and at 3, 6 and 12 months.

▪ Intensity of Pain

Seven of the studies reflected a significant improvement with regard to pain, represented in all of them through the Visual Analogical Scale (VAS). Nevertheless, the inclusion of DN did not show relevant differences concerning the intensity of pain at any time during the follow-up period. The study conducted by Imani et al. [30] also used the numerical Pain Rating Scale (NPRS), which is similar to the VAS scale.

Ekici et al. [33] recorded night pain, pain at rest and pain during activity, highlighting differentiating effects between groups at the end of the study (12 months’ follow-up) in favor of the EG—confirming a significant reduction in night pain compared to the other groups.

▪ Range of Movement

The results concerning ROM (measured by goniometry) varied widely between studies. Halle et al. [32] reported an improvement in shoulder flexion in favor of the control group (conventional physiotherapy). Jalilipanah [26] observed significant differences (*p* < 0.001) in shoulder abduction (ABD) in the groups that included DN in the treatment. Koppenhaver et al. [27] evaluated horizontal shoulder adduction (ADD) and internal shoulder rotation. Ekici et al. [33] demonstrated significant differences (*p* < 0.05) in relation to internal shoulder rotation throughout the follow-up, but especially after one-year post treatment in the group that received DN.

▪ Pain Pressure Threshold

In [25,26,27,30], the assessment of pressure pain in the treated muscles was carried out by pressure algometry, in all cases using different models of the Wagner device. In all groups, good results were observed, with significant differences post-treatment—especially in the ISP muscle [25]—but between groups the improvements were not relevant. DN is an applicable technique which can synergistically decline pain when it is combined with manual techniques to treat shoulder dysfunctions [31].

▪ Functional Assessment

The DASH questionnaire [36] was used by six of the studies to put the progression of functionality in objective terms depending on the treatment. Kamali et al. [25] found no significant differences between groups, unlike Arias-Buría [16], who did find significant differences in all the periods (immediately, 3–6 and 12-months post-intervention) between the CG and EG, confirming an improvement in functionality. Kheradmandi et al. [31] found significantly reduced values on the DASH questionnaire and pain in both groups after treatment.

Koppenhaver and Jalilipanah [26,27,28,29,30,31,32,33] used the Penn Shoulder Score (PSS), revealing significant improvements eight days’ post-intervention with respect to the basal measurement (*p* < 0.01), but nothing relevant between study groups.

Additionally, Halle and Imani [30,32] opted for scales such as the GROC (Global Rating of Change functional outcome score) and SPADI (Shoulder Pain and Disability Index functional outcome measure), where the trend of all the studies was followed as a significant improvement was observed during the application of the treatment, but no differences were discerned between groups—with the exception of EG_1_ (deep dry needling) in the study by Imani, in which significant differences were found in the SPADI scale.

▪ Cost–Benefit Ratio

Highly relevant data can be derived from the study by Arias-Buría et al. [29], who used scales such as the EuroQol-5D (quality of life scale) and QUALY (Quality-Adjusted Life-Year) scale measured at baseline and at the end of the study (12 months)—a duration that endorses the relevance of the results, with statistically significant differences observed between the CG and EG (*p* < 0.001) in favor of the EG, which recorded a better cost-effectiveness ratio of treatment and greater quality of life.

The analysis of indirect costs focused on absenteeism from work. The subjects in the CG missed 805 workdays compared to those in the EG with only 56. The EG presented with 60% less absenteeism, representing lower social costs, which were 77% higher in the CG (lack of productivity and consumption of public resources).

### 3.4. Characteristics of the Interventions

Two studies [27,31] did not have any comparison branches, five of the studies [25,28,29,32,37] had two comparison branches, and two of them [26,30] had three comparison branches. The studies compared DN therapy with an exercise program [28]; a physiotherapy routine (interferential current, hot pack and some exercises) [30]; manual therapy [31]; standard rehabilitation protocol PROM [32]; TrPs deep friction massage [33]; Muscle Energy Technique [26]; and DN in UT or ISP muscles [25].

The types of DN techniques used were manual interventions, varying among the studies: “Sparrow technique” (in and out motion) [27]; Hong’s DN technique [28,29,30]; DN active TrPs [25,26,30]; pistoning technique (inserting and withdrawing needle rapidly from each TsPs; needling with electrical stimulation [32]; and fast input/output technique [33].

With regard to the number and length of sessions, the studies were very heterogeneous. Ekici et al. conducted six sessions over a four-week period, with a treatment every five days [33]; three studies conducted four sessions (during the second/fourth treatment session) once per week [28,29,32]; four of the studies conducted three sessions: two of them in a one-week period with at least a two-day break between sessions [25,26], another with intervals of three days [31], and one study during the third, fifth and seventh sessions [30]; and finally, one study conducted only one session of treatment.

In studies where specify the time, the duration of DN therapy was conducted for 5–10 min [27,28,29] (Table 5).

## 4. Discussion

Different studies have confirmed the effectiveness of the DN technique by comparing it to the application of a placebo, effectively reducing pain in four weeks in different pathologies [39,40,41]—especially in the upper limb, and even with a single session of DN [37].

Different articles [8,42] have concluded that there very little evidence actually exists in regard to the treatment of shoulder dysfunctions, and that more studies are necessary in order to clarify the effects of DN—especially in the infraspinatus muscle, where it is the most effective method [25].

In the clinical trial conducted by Arias-Buría et al. [16], the effects of TE were compared in combination with or isolation from DN; a protocol of TE that consisted of carrying out three exercises focused on the SSC, ISP, and scapula stabilizing musculature for four weeks was carried out once a week with a duration of 25 min.

Actually, it is surprising that patients were able to obtain a significant improvement, given the very small stimulus to which the treatments with TE were reduced, since according to Izquierdo [43] it is highly unlikely that significant improvements would be produced for people with functional limitations with a training frequency of less than two days a week. Hence, in order to really consider an acceptable comparison between a therapy with DN and another with TE, the latter must be accepted by the current standards on muscle performance.

Along these lines, the American College of Sports Medicine (ACSM) [44] recommends that the training session for people with functional limitations should have a duration of at least 45 min, 20 more than what was put forward in this study initially. Current scientific evidence in relation to exercise and soft tissue injuries in the shoulder is as yet limited [45].

The most promising study for future reviews is the one by Hando et al. [35], which has a greater sample (*n* = 130) and includes a particular blinding method that is highly suitable for this procedure, with an experimental group treated with ‘Sham Dry Needling’, which improves the reliability of the results—which are expected to be released this year (the main results) and mid 2023 (the secondary results).

Through this SR, the positive cost–benefit ratio of the application of DN is verified. The extracted data show a clear difference in terms of the effectiveness of the DN technique in SAPS compared to conventional physiotherapy treatment. The results are long-lasting, which will prevent relapses and will improve the cost derived from the treatment of SAPS. 

Although the effectiveness of the interventions implies an improvement in functional limitations, this is not related to the effectiveness of the technique in terms of the ability to work or the duration of sick leave [46]. It is recommended that the efficacy of DN in other pathologies be investigated to confirm its usefulness, and thereby add it to other treatments that are carried out in clinical practice.

The DASH scale, the most widespread questionnaire and the one with the lowest absolute measurement error, or the SPADI, recommended in patients whose treatment requires surgery, have proven to be an objective, valid, safe tool applicable to a large number of pathologies [47]—comparable to other more commonly used scales such as the VAS and other health status measurements [22].

Indeed, DN may be one of the most useful and often-studied invasive physical therapy applications in musculoskeletal disorders of different body regions (included shoulder arm) and multiple soreness location disorders [48].

## 5. Conclusions

DN has been demonstrated to be effective at reducing the pain and disability produced by SAPS, obtaining the best results in combination with conventional physiotherapy rather than as an isolated technique.

The results obtained reflect an improvement in all cases of pain-related shoulder injuries, with the best combination being the application of DN together with conventional physiotherapy (TE, TM)—also achieving more stable, longer-lasting benefits than application of these techniques in isolation.

Nevertheless, as for a gain in ROM, the results suggest that no significant differences are produced, although in no case did its application turn out to be counterproductive.

Finally, all the studies point towards the application of the technique in the ISP muscle as being the most effective way of obtaining satisfactory results. We hope this SR will stimulate researchers to further explore the mechanisms and effects of DN by conducting experiments that are both methodologically sound and clinically relevant.

## Figures and Tables

**Table 1:** Measures used to assess results and effects.

VARIABLES
Pain: VAS, NPRS
Function: PSFS, ROM, PSFS, scapular dyskinesia, infraspinatus muscle function.
Shoulder assessment: DASH, PSS, GROC, SPADI.
Quality of life: Euro-QoL-D5, QALY
Muscular sensitivity: PPT, pressure algometer

**Table 2:** Methodological quality review of the included studies using the PEDro evaluation scale.

PEDro Criterion	Arias-Buría et al. [16]	Arias-Buría et al. [17]	Kheradmandi et al. [19]	Imani et al. [18]	Kamali et al. [23]	Halle et al. [20]	Ekici et al. [21]	Jalilipaanah et al. [22]	Koppenhaver et al. [15]
1 *	1	1	1	1	1	1	1	1	1
2	1	1	1	1	1	1	1	1	1
3	1	1	1	1	1	1	1	1	-
4	1	1	1	1	1	1	1	1	1
5	1	1	1	1	-	1	-	-	-
6	1	1	1	1	-	1	1	-	1
7	1	1	1	1	1	1	1	-	-
8	1	1	1	1	1	1	1	1	1
9	1	1	-	-	-	1	1	-	1
10	1	1	1	1	1	1	1	1	1
11	1	1	1	1	1	1	1	1	1
Total	10/10	10/10	9/10	9/10	7/10	10/10	9/10	6/10	7/10

Criterion in the PEDro scale: 1 = eligibility criteria; 2 = random allocation of subjects; 3 = allocation concealed: 4 = baseline comparability of important measures; 5 = blinding of subjects; 6 = blinding of therapists; 7 = blinding of assessors; 8 = measures obtained for >85% subjects; 9 = intention to treat analysis; 10 = between-group statistical comparisons; 11 = point measures and measures of variability. * Does not contribute to the total PEDro score. A score of ‘1′ indicates that the criterion is met while a score of ‘-’ indicates that the criterion is not met.

**Table 3:** Assessment of studies using the JADAD scale.

JADAD SCALE							TOTAL
Author	Title	1	2	3	4	5	Σ
Arias-Buría J.L., et al., 2017 [28]	Exercises and Dry Needling for Subacromial Pain Syndrome: A Randomized Parallel-Group Trial. *J pain*. 2017;18(1):11–18. doi:10.1016/j.jpain.2016.08.013	1	1	0	1	1	4
Arias-Buría J.L., et al., 2018 [29]	Cost-effectiveness Evaluation of the Inclusion of Dry Needling into an Exercise Program for Subacromial Pain Syndrome: Evidence from a Randomized Clinical Trial. *Pain Med*. 2018;19(12):2336–2347. doi:10.1093/pm/pny021	1	1	0	1	1	4
Kheradmandi A., Kamali F., Ebrahimian M., Abbasi L. 2021 [31]	Comparison between dry needling plus manual therapy with manual therapy alone on pain and function in overhead athletes with scapular dyskinesia; A randomized clinical trial. J Bodyw Mov Ther. 2021 Apr:26:339–346. Doi:10.1016/j.jbmt.2020.11.017. Epub 2020 Nov 24. PMID: 33992267.	1	1	0	1	1	4
Imani M., Abbasi L., Taghizadeh S., Amiri M., 2021 [30]	Comparison of the effect of two different types of dry-needling techniques on subacromial impingement syndrome. *J Bodyw Mov Ther*. 2021;25:35–40. doi:10.1016/j.jbmt.2020.10.018	1	1	0	1	0	3
Kamali F., et al., 2019 [25]	Comparison of Upper Trapezius and Infraspinatus Myofascial Trigger Point Therapy by Dry Needling in Overhead Athletes With Unilateral Shoulder Impingement Syndrome. *J Sport Rehabil*. 2019;28(3):243–249. doi:10.1123/jsr.2017-0207	NA	NA	NA	NA	NA	NA
Halle R, Crowell M, Goss D., 2020 [32]	Dry needling and physical therapy versus physical therapy alone following shoulder stabilization repair: a randomized clinical trial. *Int J Sports Phys Ther*. 2020;15(1):81–102.	1	1	0	1	1	4
Ekici G, Özcan Ş, Öztürk BY, Öztürk B, Ekici B., 2021 [33]	Effects of Deep Friction Massage and Dry Needling therapy on Night Pain and Shoulder Internal Rotation in Subacromial Pain Syndrome: 1-year Follow up of a Randomised Controlled Trial. *Int J Ther Rehabil*. 2021;28(2):1–12. doi:10.12968/ijtr.2020.0018	1	1	0	1	1	4
Jalilipanah P., Okhovatian F., Serri R.A., Bagban A.A., Zamani S., 2021 [26]	The effect of Dry Needling and Muscle Energy Technique Separately and in Combination in Patients Suffering Shoulder Impingement Syndrome and Active Trigger Points of Infraspinatus. *J Bodyw Mov Ther*. 2021; 26:94–100. doi:10.1016/j.jbmt.2020.12.030	1	1	0	1	0	3
Koppenhaver S., et al., 2016 [27]	Effects of Dry Needling to the Symptomatic versus Control Shoulder in Patients with Unilateral Subacromial Pain Syndrome. *Man Ther*. 2016;26:62–69. doi:10.1016/j.math.2016.07.009	NA	NA	NA	NA	NA	NA

NA: not applicable.

**Table 4:** Characteristics of the studies selected.

References	Participant Profile	Intervention	Follow-Up	Variables	Results	Conclusions	Adverse Effects and Limitations
Koppenhaver S., et al., 2016 [27]	*n* = 57 Female: *n* = 35.7% Male: *n* = 64.3% Age 44.1 ± 10.1 yo Department of Defense beneficiaries, from Joint Base San Antonio, Texas. Individuals who would seek healthcare for unilateral SAPS without any contraindications to DN.	DN in ISP muscle	Duration: 1 W Measurements: Basal immediately after treatment.	- p-PENN scale - IMF - Shoulder ROM - Pressure algometry	- PENN Scale: 60.1% + S.I. - IMF: no significant differences. - ROM: *p* < 0.01; S.I. - Pressure algometry: *p* = 0.01. S.I. Losses (*n* = 10)	Found changes in shoulder ROM and pain sensitivity, but not in muscle function, after DN. These changes generally occurred 3–4 days after DN and only in the symptomatic shoulders.	No adverse effects or pain.
Arias-Buría J.L., et al., 2017 [28]	*n* = 50 (EG: 25/CG: 25) Female: *n* = 6 Male: *n* = 19 Age ± 48 yo Consecutive subjects with diagnosis of SAPS from a Spanish regional hospital.	EG: TE CG: TE + DDN	Duration: twice a day for 5 W Measurements: Basal: - 1 W after the last treatment - 3/6/12 M after end of treatment.	- Disabilities of the arm, shoulder and hand, DASH questionnaire - PI	DASH: S.I. in EG, 1 week after, at 3/6 and 12 M compared to the CG (*p* < 0.001) PI: Improvement in both groups. No significant differences in worst pain (*p* = 0.43) Losses (*n* = 3): CG (*n* = 2) EG (*n* = 1)	The inclusion of two sessions of TrP-DN into a TE program was effective for improving shoulder pain-related disability in the short, medium, and long term	No greater improvement in shoulder pain was observed.
Arias-Buría J.L., et al., 2018 [29]	*N* = 50 (EG: 25/CG:25) Female: *n* = 13 Male: *n* = 37 Age EG 48 ± 5 yo Age CG 49 ± 4 yo Patients with unilateral nontraumatic shoulder pain of at least 3 M duration and PI of at least 4 points on an 11-point NPRS, from an urban hospital in Madrid, Spain.	EG: Exercise program + TrP-DN CG: Exercise program alone	Duration: twice a day for W Measurements: Basal, 1/3/6 and 12 M	EuroQol-D5	EuroQol-D5: Greater number of visits to orthopedic surgeon and greater number of treatments added in CG (*p* < 0.001) Statistically significant differences in relation to absenteeism from work which is greater in CG (*p* = 0.001) Greater quality of life in EG (+2.87 QALY) Cost-benefit: EUR −12,933.54 per year in EG Losses (*n* = 0)	The inclusion of TrP-DN into an exercise program was more cost-effective for individuals with SAPS than exercise alone.	No greater improvement in shoulder pain was observed.
Imani M., Abbasi L., Taghizadeh S., Amiri M., 2021 [30]	*n*= 66 (EG_1_ = EG_2_ = CG: 22) Female: *n* = 42 Male: *n* = 24 Age 43.24 ± 9.961 yo Patients with shoulder impingement syndrome.	EG_1_: TE + E + TM + SDDN EG_2_: TE + E + TM + HDDN CG: TE + E + TM	Duration: nine physiotherapy sessions Measurements: Basal immediately after, and at 4 W	- SPADI - NPRS - Digital pain pressure algometer (Wagner RS232)	PI: Significant reduction in all groups immediately after treatment (*p* = < 0.05), positive trend maintained for EG_1_ Level of disability: S.I. in EG_1_ at the end of the study (*p* < 0.05) Losses (*n* = 8): CG (*n* = 2), EG_1_(*n* = 3),EG_2_ (*n* = 1)	There was no significant difference between the three groups in pressure pain tolerance threshold and pain reduction.	No greater improvement in shoulder pain was observed.
Kheradmandi A., Kamali F., Ebrahimian M., Abbasi L. 2021 [31]	*n* = 40 Female: *n* = 25 Male: *n* = 15 (EG: 20/CG:20) Age: 18–45 yo Athletes with SD With at least three points NRS PI during training	EG: MT + DN in TP of Subescapularis, pectoralis minor, Serratus anterior, upper and lower Trapezius muscles CG: MT alone	Duration: three sessions with the interval of every 3 days.	- Effect of shoulder TP DN + MT with MT alone on: - Pain - Function - PPT - SD	Pain, disability and SD were improved in EG (*p* < 0.05) CG: reduction in pain and disability (*p* < 0.001). Scapular slide only improved in hands on waist position. Comparing the differences between groups improvement in SD in EG (*p* = 0.02). PPT significantly increased in the CG (*p* = 0.004). Losses (*n* = 0)	DN is an easy and applicable method that can synergistically reduce pain, disability and SD when it is combined with MT techniques to treat shoulder dysfunctions.	No adverse effects reported by the participants.
Halle R, Crowell M, Goss D., 2020 [32]	*N* = 39 (EG: 19/CG: 20) Female: *n* = 6 Male: *n* = 34 Age 20.78 ± 3.33 Post-operative shoulder patients.	EG: SPP + HDDN CG: SPP	Duration: 6 M Measurements: 4/8/12 W, and 6 M post-operation	- Glenohumeral articulation ROM - NPRS - GROC - PSFS - SPADI	No significant differences, except in shoulder flexion in CG (*p* = 0.019)	DN in a postsurgical population is safe.	Without significant risk of iatrogenic infection or other adverse events.
Ekici G, Özcan Ş, Öztürk BY, Öztürk B, Ekici B., 2021 [33]	*n* = 40 (EG_1_: 19/EG_2_: 21) Female: *n* = 31 Male: *n* = 18 Age EG_1:_ 50.90 ± 7.88 yo Age EG_2_: 52.04 ± 8.98 yo Outpatients diagnosed with SAPS.	GE_1_:TrP Deep friction massage GE_2_: TrP-DN	Duration: 4 W (six physiotherapy sessions) Measurements: Basal: 4 W, 12 M	- Duration and PI (VAS) - Active internal rotation of shoulder	Improvement in all parameters measured, but with NO significant differences between groups except for internal rotation of shoulder after 12 M follow-up (*p* < 0.05) in favor of the EG and reduction of night pain. Losses (*n* = 19): CG (*n* = 11) EG (*n* = 8)	Both interventions produced good results, TrP deep friction massage treatments were completed in a shorter time and so demonstrated earlier improvements. TrP deep friction massage may be regarded as the preferred option, particularly as no equipment is needed and it is a non-invasive method of treatment.	No adverse effects reported by the participants.
Jalilipanah *p*., Okhovatian F., Serri R.A., Bagban A.A., Zamani S., 2021 [26]	*n* = 39 (EG_1_ = E_2_ = EG_3_: 13) Age: 20–50 yo Patients with shoulder impingement Syndrome and active TrP in the ISP muscle.	EG_1_: HDDN EG_2_: Post-isometric relaxation GE_3_: HDDN + post-isometric relaxation	Duration: 1 W Measurements: Basal	- PI - PPT - ROM - PSS	PI: Improvement in all groups, NO significant differences between groups. PPT: Improvement in all groups, NO significant differences between groups Shoulder ROM: Improvement in all groups, Significant differences in EG_1_ and EG_2_ compared to EG_2_ Losses (*n* = 0)	Both techniques are effective in the treatment of TrP. DN is more effective in enhancing the ROM of flexion and abduction.	No adverse effects reported by the participants.
Kamali F., et al., 2019 [25]	*n* = 40 Females: *n* = 20 Males: *n* = 20 (EG_1_: 21/EG_2_:19) Age: 36 ± 16 yo Overhead athletes.	EG_1_: HDDN in descending Trapezius EG_2_: DDN in Infraspinatus	Measurements: Basal and 3 days after treatment	- PI (VAS) - PPT - DASH	PI: SI in both groups. PPT: SI in EG_2_ (*p* = 0.02), although NO significant difference between groups. DASH: NO significant differences. Losses (*n* = 6)	Application of DN for active MTrPs in the ISP can be as effective as direct DN of active MTrPs in the UT in improving pain and disability in athletes with SP, and may be preferred due to greater patient comfort in comparison with direct UT needling.	No adverse effects reported by the participants.

W = week; ISP = Infraspinatus; PENN = Disability Scale and Shoulder Pain; IMF = Infraspinatus muscle function; ROM: Range of motion; S.I. = Significant Improvement; EG = Experimental Group; CG = Control Group; SAPS = Subacromial Pain Syndrome; TE = Therapeutic Exercise; DDN = Deep DN; M = months; PI = Pain Intensity; E = Electrotherapy; TM = Thermotherapy; SDDN = Statis Deep Dry Needling; HDDN = Hong’s Deep Dry Needling; SPADI = Shoulder Pain and Disability Index; yo = Years Old; NPRS = Numeric Pain Rating Scale; SD = Scapular Dyskinesia; NRS = Numeric Rating Scale; TrP = Trigger Points; MT = Manual Therapy; PPT = Pain Pressure Threshold; MET = Muscle Energy Technique; SPP = Standard Physiotherapy Protocol; GROC = Global Rating of Change Functional Outcome Score; PSFS = Patient Specific Functional Scale; PSS = Pen Shoulder Score; MTrPs = Myofascial Trigger Points; UT = Upper Trapezius; SP = Shoulder Pain.

**Table 5:** Intervention characteristics of DN groups.

Author/Year	*n*/Number of Therapeutic Groups	Type	Time Per Session	Number of Sessions	Length of Intervention	Observations
Koppenhaver S., et al., 2016 [27]	57/1	DN technique used disposable 0.25 × 40 mm stainless Steel Seirin J-type needles. Treatment location was standardized for each participant. Needles were inserted into three general locations (superior, medial, inferior) in each ISP based on prior research and depictions of common locations of MTrP. Prior to needle insertion, manual palpation of the ISP was performed to localize treatment to the most painful area at each of the three locations. Each needle insertion lasted approximately 5–10 s, using a “sparrow pecking” (in and out motion) technique in an attempt to elicit as many local twitch responses as possible.	5 min	One	1 D	No statistically significant changes found in either resting or contracted infraspinatus muscle function in either shoulder at any time point.
Arias-Buría J.L., et al., 2017 [28] *	50/2	The protocol included the same exercise program. Each exercise was performed in three sets of 12 repetitions; each repetition included a concentric phase after the eccentric phase of the exercise. First session was taught by an experienced physical therapist and monitored in the subsequent four sessions. The program consisted of three exercises focusing on the SSP, ISP, and scapular stabilizer musculature. The TrP-DN group also received TrP-DN to active TrPs in shoulder muscles that had pain or showed shoulder symptoms during the second and fourth treatment sessions. Participants received TrP-DN with disposable stainless steel needles of 0.32mm × 40mm (Novasa, Madrid, Spain) that were inserted into the skin over the TrP. Fast-in and fast-out technique as described by Hong was applied.	20–25 min (Exercise program) 5–10 min (TrP-DN intervention)	Exercise program, on an individual basis, twice daily for 5 weeks TrP-DN: four sessions (during the second and fourth treatment sessions) once per week.	5 W	The current trial suggests that TrP-DN can be clinically used for improving the effects of exercise programs in people with subacromial pain syndrome.
Arias-Buría J.L., et al., 2018 [29] *	50/2	The protocol included the same exercise program. Each exercise was performed in three sets of 12 repetitions; each repetition consisted of a concentric phase after the eccentric phase of the exercise. The first session was taught by a physical therapist, monitored in the subsequent four sessions. The program consisted of three exercises focusing on the SSP, ISP, and scapular stabilizer musculature. TrP-DN group also received TrP-DN to active TrPs in shoulder muscles that had pain or showed shoulder symptoms during the second and fourth treatment sessions. Participants received TrP-DN with disposable stainless dteel needles of 0.32 mm x 40 mm (Novasa, Madrid, Spain) that were inserted into the skin over the TrP. Fast-in and fast-out technique as described by Hong was applied	20–25 min (Exercise program) 5–10 min (TrP-DN intervention)	Exercise program, on an individual basis, twice daily for 5 weeks TrP-DN: four sessions, (during the second and fourth treatment sessions) once per week.	5 W	NA
Imani M., Abbasi L., Taghizadeh S., Amiri M., 2021 [30]	66/3	The protocol included the same routine physiotherapy: 20 min interferential current (50–120 Hz; NOVIN Co, Multisti, 735X) with a hot pack and some exercises. DN was performed only for active TrPs and then released for 10 min to induce a local reaction. Hong’s DN technique + routine physiotherapy. Hong’s method, the needles were moved pyramidally and removed immediately after the local response appeared.	±5min	10 sessions routine physiotherapy 3 sessions of DN/Hong DN (performed in the third, fifth and seventh sessions)	4 W	NA
Kheradmandi A., Kamali F., Ebrahimian M., Abbasi L. 2021 [31]	40	The protocol included scapular mobilization (three sets of 10 repetitions with 30 s rest between each set) DN: the patients received treatment on the Subscapularis, Pectoralis minor, Serratus anterior, UT and LT.	NA	Three sessions with intervals of 3 D.	NA	DN plus manual therapy is more effective at improving pain function than manual therapy alone. Improving dyskinesia helps overhead athletes have a functional and painless workout.
Halle R, Crowell M, Goss D., 2020 [32]	39/2	The protocol included standard rehabilitation, protocols: manual PROM into flexion, abduction, external rotation, and internal rotation. DN treatment: needling techniques utilized included pistoning (inserting and withdrawing needle rapidly from each TPs), needle left in situ for 10 to 15 min, needling with electrical stimulation, and a combination of these techniques.	Equal amounts of time both groups	Weekly DN (four treatments)	4 W	NA
Ekici G, Özcan Ş, Öztürk BY, Öztürk B, Ekici B., 2021 [33]	40/2	A protocol including TrP deep friction massage was applied transversely and deeply, following the fibre direction of the affected connective tissue, until analgesia occurred. TrP-DN therapy: the needle type and depth of placement were changed according to the estimated muscle thickness (0.25–25 mm, 0.25–30 mm, 0.25–40 mm). A fast input/output technique was preferred for the TrPs, through the taut band of the muscle. The process was continued until no more local twitch response was achieved. Movements were in the vertical direction between 3 and 5 mm.	NA	Six sessions, twice a week over a 3-week period. DN group received six treatments over a 4-week period, with a treatment every 5 D.	4 W	Both groups received six treatment sessions and a programme of post-treatment exercises.
Jalilipanah P., Okhovatian F., Serri R.A., Bagban A.A., Zamani S., 2021 [26]	39/3	MET: PIR, treatment administered according to L. Chaitow’s guidelines [38]. DN: needles were inserted directly into the muscle, then partly removed and then re-inserted; this process was repeated until no further local twitch responses were elicited. The treatment was done with a 25 mm, 0.25 G acupuncture needle.	NA	Three sessions in a one-week period, with at least a 2 D break between sessions.	1 W	DN was more effective than MET and their combination in enhancing the abduction and flexion ROMs. In healthy subjects, we have to note that combinations of the DN and MET methods can be equally effective with latent TrPs. The most effective method is the one that can quickly reduce pain and enhance the ROM.
Kamali F., et al.,2019 [25]	40/2	A protocol with DN was applied directly onto TrPs in the UT. The needle should be inserted perpendicular to the skin, toward the therapist’s finger. Indirect DN needling in the ISP; direct needling into TrPs towards the scapula while the patient lay in the prone position. In both techniques a 0.2 × 50 mm acupuncture needle with a guiding tube was used.	NA	Three sessions (2 D intervals between sessions)	2 W	Patients were not allowed to receive any drug or other type of treatment during the trial.

DN: Dry Needling; MTrP: Myofascial Trigger Points; UT: Upper Trapezius Muscle; SSP: Supraspinatus Muscle; ISP: Infraspinatus Muscle; D: Day; W: Week; TrPs: Trigger Points; TrP-DN: Trigger Point Dry Needling; LT: Lower Trapezius; MET: Muscle Energy Technique; PIR: Post-Isometric Relaxation; ROM: Range of Movement. * These publications belong to the same study, but evaluate different outcomes. N Therapeutic Group: Patients allocated in number of groups.

## Data Availability

Not applicable.

## Appendix A

**Table A1:** Search strategy in databases.

Databases	Search Strategy
PUBMED (up to current date, 2021) (SR no limit date; RCTs from 2016 until 16 December 2021)	[no MeSH terms]: “Dry needling” AND “Shoulder pain” AND “Physiotherapy” [no MeSH terms]: “Dry” AND “needling” AND “shoulder” AND “pain” AND “physiotherapy”
SCOPUS (up to current date, 2021) (SR no limit date; RCTs from 2016 until 16 December 2021)	I./II. [no MeSH terms]: “Dry” AND “needling” AND “Shoulder” AND “pain” AND “physiotherapy”
Web of Science (up to current date, 2021) (SR no limit date; RCTs from 2016 until 16 December 2021)	I./II. [no MeSH terms]: “Dry” AND “needling” AND “Shoulder” AND “pain” AND “physiotherapy”
PEDro (up to current date, 2021) (SR no limit date; RCTs from 2016 until 16 December 2021)	I./II. [no MeSH terms]: “Dry” AND “shoulder”
Cochrane Library (up to current date, 2021) (SR no limit date; RCTs from 2016 until 16 December 2021)	I./II. [MeSH tems]: “Impigement syndrome” AND “needles”
Tripdatabase (up to current date, 2021) (SR no limit date; RCTs from 2016 until 16 December 2021)	I./II. [no MeSH terms]: “Dry” AND “Needling” AND “Shoulder” AND “pain” AND “physiotherapy”
